# Adolescent Attitudes Toward the Human Papillomavirus (HPV) Vaccine in Kocaeli, Turkiye

**DOI:** 10.7759/cureus.78760

**Published:** 2025-02-09

**Authors:** Melisa Y Gundogdu, M Z Gezer, Zuhal Gundogdu

**Affiliations:** 1 Research, Nesibe Aydin Kocaeli, Kocaeli, TUR; 2 Faculty of Medicine, Kocaeli University Hospital, Kocaeli, TUR; 3 Pediatrics, Kocaeli University, Kocaeli, TUR

**Keywords:** adolescents, attitudes, beliefs, cancer, hpv, human papillomavirus, vaccine

## Abstract

Background

The adolescent human papillomavirus (HPV) vaccination rate is relatively high in developed countries and it is not widespread in Turkiye. This paper explores what factors influence the decision on the acceptance of the HPV vaccine by adolescents.

Methods

Data from 206 adolescents was gathered via a survey completed after consent from the parents and themselves between May 2022 and September 2022. The survey content was based on the Carolina HPV Immunization Attitudes and Beliefs Scale (CHIAS), each question being based on a 5-point Likert scale, together with additional questions to discover demographic factors. The participants were also questioned about their level of knowledge of HPV and its source. At the end of the questionnaire, once the adolescents were briefed on HPV vaccination by a doctor, questions about immunization against HPV were again redirected.

Results

The age of 206 participants was between 12 and 26 years old, and it was found that the knowledge level about the HPV vaccine increases as the age gets older. The adolescents believe that HPV protects more from cervical cancer than genital warts. 8.7% (n=18) of adolescents thought that the HPV vaccine could have long-term adverse effects. 33.5% (n=69) of them stated that their families could not afford the vaccine. After they were given more knowledge, it was found that they were more willing to have HPV immunization.

Conclusions

It is important to realize that teenagers' opinions can shift significantly, particularly in the context of informed decision-making and the provision of government financial assistance to parents. The comprehension of teenagers' perspectives and the extent of their knowledge regarding HPV is of paramount importance, given the likelihood of it being influenced by their own acceptance of the vaccine.

## Introduction

Human papillomavirus (HPV) remains a major source of morbidity and mortality. HPV is usually a sexually transmitted infection linked to cancers such as cervical cancer, head and neck squamous cell carcinoma (HNSCC), and anal cancer [1-3]. However, despite this, acceptance of vaccines depends on the perception of the seriousness of the disease [4]. To date, more than 200 HPV types have been identified [5-7].

Different strains of HPV target the mucosal tissues of the oral and cervical tracts, whereas others infect cutaneous tissues and cause warts [7-9]. HPV16, 18, 31, and 33 are among the mucosal HPV types that are classified as either high-risk HPV or oncogenic HPV types based on their ability to cause cancer. The majority of low-risk, non-oncogenic HPV strains (HPV6 and 11) are identified in warts [7,9-10].

Three HPV vaccines have been developed to protect against up to nine HPV types. These vaccines are highly efficient at preventing cervical infections caused by these HPV strains, as well as condylomas and several HPV-related malignancies [7].

The quadrivalent HPV vaccine Gardasil (Merck & Co., Rahway, NJ, USA) was the first HPV vaccine approved by the FDA in 2006. Besides HPV16 and 18, it also targets HPV6 and 11, which cause 90% of genital warts [11]. The bivalent HPV vaccine, Cervarix (GSK, Brentford, UK), was approved by the EMA in 2007 and the FDA in 2009 [7,12]. Cervarix protects against HPV types 16 and 18, responsible for 70% of cervical cancers [7,13]. In 2014, the nine-valent vaccine Gardasil 9 (Merck & Co.) was licensed by the FDA, offering protection against HPV6, 11, 16, 18, 31, 33, 45, 53, and 58. Gardasil 9 can potentially protect against approximately 90% of cervical cancers by covering additional HPV types linked to another 20% of cases [7,13-14].

To eradicate cervical cancer as a public health issue, the World Health Organisation (WHO) is creating a global strategy that calls for an elimination threshold of four cases per 100,000 women. The strategy also includes triple-intervention coverage targets for 2030, which include increasing the number of HPV vaccinations to 90%, twice-lifetime cervical screening to 70%, and treating pre-invasive lesions and invasive cancer to 90% [11].

The WHO strongly recommends HPV vaccination for girls aged nine to 14 years, recognising it as an essential and cost-effective public health policy [15]. Numerous studies have therefore assessed adolescents' attitudes towards HPV vaccination [16-20].

It is evident that the majority of low- and middle-income countries are not adequately protected, with only approximately 1% of adolescent females in low-income countries receiving a full course of HPV vaccines [7,21]. As of November 2024, the HPV vaccine is not included in the national vaccination program in Turkiye, and it is not a vaccine that can be accessed by everyone due to its cost.

Our study focused on obtaining comprehensive insights into HPV and its vaccination in adolescents. The primary objective was to investigate the attitudes and knowledge of adolescents, as well as to identify the determinants influencing their decision-making regarding HPV vaccination using the Carolina HPV Immunisation Attitudes and Beliefs Scale (CHIAS). This study is intended to enhance the uptake of HPV vaccination among adolescents.

## Materials and methods

Data were gathered from 206 participants in Kocaeli, Turkiye, between May 2022 and September 2022, following a post-consent survey that parents of teenagers under the age of 18 completed. The participants, who ranged in age from 12 to 26, were chosen at random from among high school and university students. Only healthy participants with no known chronic illnesses were included.

The content of the survey was based on eight questions adopted from CHIAS [3], with each question based on a 5-point Likert scale, and a further 11 questions.

The purpose of the questionnaire was to gather information about the adolescents' HPV vaccination status and HPV dose in addition to sociodemographic details (date of birth, sex, family income, parents' educational level, age and number of siblings). The level and source of knowledge about HPV was also asked about.

At the end of the survey, participants were given more knowledge about HPV and HPV vaccines through a written information sheet as well as face-to-face. After this, they were asked if would accept the HPV vaccine or not, and their responses were recorded.

This study was approved by the Non-Invasive Clinical Research Ethical Committee of Kocaeli University GOKAEK-2022/05.06. 

Analytic strategy

The survey content utilized health belief statements from the CHIAS for the HPV vaccine [3]. Participants were asked to indicate their level of agreement with each statement by using a 5-point Likert scale, ranging from "strongly agree" to "strongly disagree". Their responses were then converted to an ordinal scale with values between 1 and 5, where 1 represents "strongly disagree" and 5 represents "strongly agree".

Analysis was performed using SPSS, version 17 (IBM Inc., Armonk, NY, USA). Frequency and percentiles were calculated and compared; the Mann-Whitney test was used to determine significance because there was no prior information on the data distribution, no parametric test was used, and the dependent variable was presented on an ordinal scale.

## Results

According to the survey, HPV vaccination knowledge increases with age among 206 participants aged 12 to 26. Eight (3.9%) adolescents were vaccinated with one dose, 5.8% (n=12) were vaccinated with two doses, and 5.8% (n=12) were vaccinated with three doses. Overall, 84.5% (n=174) of adolescents did not receive any HPV vaccination. However, 71.8% (n=148) of participants were aware of HPV vaccines.

The participants who were aware of the HPV vaccine reported that 22.8% (n=36) of their fathers had college degrees and 52.6% (n=83) had university degrees, while 24.7% (n=39) of their mothers had college degrees and 36.1% (n=57) had university degrees. Table 1 presents the respondents' social and demographic characteristics. A comparison between adolescents who are aware of the HPV vaccine and those who are not show a statistically significant difference (p < 0.05). It is also evident that participants with older age group parents especially between the age of 45-54 are more aware of the HPV vaccine.

**Table 1:** Several demographic factor frequencies regarding the human papillomavirus (HPV) vaccine from a study carried out in Kocaeli, Turkiye between May and September 2022.

Demographic factors	HPV vaccine knowledge (Yes), N:148 (71.8%)	HPV vaccine knowledge (No), N:58 (28.1%)	p
Age of participants	21.93+/-2.93	18.43+/-3.50	0.005
Father's age (in years)			<0.001
35–44	5 (3.2%)	11 (22.9%)	
45- 54	98 (62%)	32 (66.7%)	
55 and above	55 (34.8%)	5 (10.4%)	
Mother's age (in years)			<0.001
35–44	34 (21.5%)	19 (39.6%)	
45- 54	102 (64.6%)	29 (60.4%)	
55 and above	22 (13.9%)		
Income			<0.001
1 (Low income)	24 (15.2%)	9 (18.8%)	
2 (Middle income)	123 (77.8%)	37 (77.1%)	
3 (Upper income)	11 (7%)	2 (4.2%)	
Father's education			0.003
1 (Primary school)	1 (0.6%)		
2 (Secondary school)	36 (22.8%)	20 (41.7%)	
3 (College)	36 (22.8%)	5 (10.4%)	
4 (University)	83 (52.6%)	23 (47.9%)	
5 (Postgraduate)	2 (1.3%)		
Mother's education			<0.001
1 (Primary school)	14 (8.9%)	3 (6.3%)	
2 (Secondary school)	46 (29.1%)	22 (45.8%)	
3 (College)	39 (24.7%)	5 (10.4%)	
4 (University)	57 (36.1%)	18 (37.5%)	
5 (Postgraduate)	2 (1.3%)		
Number of siblings			0.02
0	4 (2.5%)		
1	70 (44.3%)	19 (39.6%)	
2	48 (30,4%)	14 (29.2%)	
3	9 (5.7%)	4 (8.3%)	
≥4	27 (17.1%)	11 (22.9%)	

The majority of participants, 79.6% (n=164), think that the HPV vaccination offers greater protection against genital warts and cervical cancer, but 39.3% (n=81) are also worried that HPV could have negative immediate effects, and 8.7% (n=18) think that more negative effects might manifest later in life (Table 2). Regarding the last statement, “Since the HPV vaccine is a new vaccine, one should wait more before getting the vaccine," 51% (n=105) of adolescents disagreed, 28.6% (n=59) neither disagreed nor agreed, and 20.4% (n=42) agreed with it (Table 2). 

**Table 2:** Adolescents’ belief regarding human papillomavirus (HPV) vaccine from a study carried out in Kocaeli, Turkiye

Statements	Agree	Neither agree nor disagree	Disagree
I think the HPV vaccine is not safe	9 (4.4%)	52 (25.2%)	145 (70.4%)
HPV vaccine could cause acute adverse effect	81 (39.3%)	103 (50%)	22 (10.7%)
HPV vaccine could cause health problems years later	18 (8.7%)	65 (31.6%)	123 (59.7%)
I am concerned that the HPV vaccine costs more than my parents can pay.	69 (33.5%)	75 (36.4.3%)	62 (30.1%)
It would be hard to find a provider or clinic where to be vaccinated.	74 (35.9%)	54 (26.2%)	78 (37.9%)
HPV vaccine is effective in preventing cervical cancer and genital warts	164 (79.6%)	41 (19.9%)	1 (0.5%)
Other teenagers around me get themselves vaccinated with HPV	31(15.1%)	66 (32.5%)	108(52.4%)
Since the HPV vaccine is new, one should wait longer before getting the vaccine	42 (20.4%)	59 (28.6%)	105 (51%)

Table 3 shows the level and source of information about HPV. While most participants, 43.6% (n=90), had heard about HPV from their teachers, 32.5% (n=67) of adolescents felt that they had enough detailed knowledge about the HPV vaccine.

**Table 3:** Source/level of knowledge about human papillomavirus (HPV) vaccine

Enough & Accurate information	A few sources	Media	Friends	Family	Health Professionals	Own College and University Teachers
67 (32.5%)	81 (39.3%)	30 (14.5%)	6 (2.9%)	6 (2.9%)	16 (7.7%)	90 (43.6%)

Upon completion of the survey, each adolescent was provided with information regarding the HPV vaccine, after which 83.5% (n=172) of the adolescents elected to receive the HPV vaccine. However, around 33.5% (n=69) of them stated that their families could not afford to pay for the vaccine.

## Discussion

These findings describe adolescents’ opinions about the HPV vaccine. Studies on vaccine attitudes and opinions were usually carried out on parents. However, only a few studies also explored adolescent attitudes towards the HPV vaccine [16-20].

Awareness of adolescent HPV vaccination was higher among female parents, those with higher levels of education, and individuals aged 35-45 years [22]. Our study shows similar results, suggesting that parents' age and education level affect their awareness of HPV vaccination.

Numerous studies indicate that women's knowledge and educational interventions are effective in preventing HPV transmission, enhancing the acceptability of the HPV vaccine, and consequently improving HPV vaccine coverage [23-25].

A number of studies have indicated that the provision of educational videos has the potential to increase the acceptance of the HPV vaccine by approximately 20% among young women [23]. Educational videos that outline the risks of HPV and the benefits of vaccination have been found to be an effective means of improving vaccination uptake [23-24].

Our study shows that 43.6% (n=90) of adolescents have been informed through their college education. Social media also plays a key role in health-related issues. The cost of the vaccine has an impact on those with a low income [24]. Cost-effectiveness is therefore one of several important factors that should be considered by decision-makers when planning a new health intervention [26]. Our study found that both the financial burden of the vaccines and the lack of quality information were key factors in HPV vaccine decision-making. Our results show that 62 adolescents (33.5%) are concerned that the HPV vaccine will cost more than their parents can afford.

Although 71.8% (n=148) of adolescents had knowledge about the HPV vaccine, only 3.9% (n=8) of adolescents had received one dose, 5.8% (n=12) had received two doses, and 5.8% (n=12) had received three doses. These results may be due to HPV vaccine costs.

As demonstrated in a number of studies, all three vaccines have been found to have excellent safety and tolerance across a diverse range of age groups [27-29]. As indicated by the literature, the most cited reasons for vaccine non-acceptability are doubts about the efficacy of the vaccine, cost, and fear of safety and side effects. This is in line with the findings from reviews of girls [5]. It is imperative to make the people understand the severity of vaccine-preventable diseases in order to facilitate acceptance of vaccines. This highlights the need for effective communication about these risks to avoid parental misconceptions about vaccine safety and efficacy [5].

One limitation of our study is that some participants were under 18 years of age, requiring parental consent. This may have introduced potential bias, as we are unsure of the extent to which parents influenced their adolescents' decisions regarding participation. Nevertheless, the number of such participants was considerably lower than the rest. Another constraint is the limited total number of participants in the study. However, the diverse socioeconomic background of Kocaeli, a cosmopolitan and industrial city, helps to mitigate this limitation by enhancing the diversity of the sample.

## Conclusions

In countries lacking government-funded immunisation programs, financial constraints and cost were major factors contributing to the decision to decline HPV vaccination. In the absence of healthcare coverage, the financial cost of the HPV vaccine may continue to be a significant barrier to acceptance and uptake in some countries, as evidenced by the case in Turkiye. The immunisation of adolescents against HPV is greatly influenced by the increased awareness regarding the risks of not having the HPV vaccine and its integration into national vaccination programs.
